# Decoding cell-type contributions to the cfRNA transcriptomic landscape of liver cancer

**DOI:** 10.1186/s40246-023-00537-w

**Published:** 2023-10-05

**Authors:** Aram Safrastyan, Christian Höner zu Siederdissen, Damian Wollny

**Affiliations:** 1 RNA Bioinformatics and High Throughput Analysis, Friedrich Schiller University Jena, Jena, Germany; 2 Leibniz Institute On Aging-Fritz Lipmann Institute (FLI), Jena, Germany; 3 Max Planck Institute for Evolutionary Anthropology, Leipzig, Germany

**Keywords:** Liver cancer, Liquid biopsy, Cellular deconvolution, Cell-free RNA, Single-cell sequencing, Modeling

## Abstract

**Background:**

Liquid biopsy, particularly cell-free RNA (cfRNA), has emerged as a promising non-invasive diagnostic tool for various diseases, including cancer, due to its accessibility and the wealth of information it provides. A key area of interest is the composition and cellular origin of cfRNA in the blood and the alterations in the cfRNA transcriptomic landscape during carcinogenesis. Investigating these changes can offer insights into the manifestations of tissue alterations in the blood, potentially leading to more effective diagnostic strategies. However, the consistency of these findings across different studies and their clinical utility remains to be fully elucidated, highlighting the need for further research in this area.

**Results:**

In this study, we analyzed over 350 blood samples from four distinct studies, investigating the cell type contributions to the cfRNA transcriptomic landscape in liver cancer. We found that an increase in hepatocyte proportions in the blood is a consistent feature across most studies and can be effectively utilized for classifying cancer and healthy samples. Moreover, our analysis revealed that in addition to hepatocytes, liver endothelial cell signatures are also prominent in the observed changes. By comparing the classification performance of cellular proportions to established markers, we demonstrated that cellular proportions could distinguish cancer from healthy samples as effectively as existing markers and can even enhance classification when used in combination with these markers.

**Conclusions:**

Our comprehensive analysis of liver cell-type composition changes in blood revealed robust effects that help classify cancer from healthy samples. This is especially noteworthy, considering the heterogeneous nature of datasets and the etiological distinctions of samples. Furthermore, the observed differences in results across studies underscore the importance of integrative and comparative approaches in the future research to determine the consistency and robustness of findings. This study contributes to the understanding of cfRNA composition in liver cancer and highlights the potential of cellular deconvolution in liquid biopsy.

**Supplementary Information:**

The online version contains supplementary material available at 10.1186/s40246-023-00537-w.

## Background

Liquid biopsy, the molecular analysis of body fluids, has emerged as a promising tool in cancer research, offering a more accessible assessment of patient health status compared to traditional tissue biopsies. Advancements in technology have enabled extensive genomic and transcriptomic analysis of DNA and RNA, especially in blood [1–4]. Among the cell-free nucleic acids being studied, cell-free RNA (cfRNA) has garnered increasing attention [5], including for its tissue and cell-type specificity [6–8].

Cellular deconvolution, a powerful computational approach, is used to determine the cellular origin of RNA in mixed transcriptomic data, such as bulk RNA-seq [9]. A multitude of cell types contributes to the formation of blood cell-free transcriptome [6], and as a result, the comparisons between such heterogeneous samples may occlude the critical differences, which are driven only by select cell types [10]. The knowledge of which specific cell types are responsible for the observed differences in the blood during, for example, carcinogenesis, will provide a more comprehensive characterization of the cell-free transcriptome perturbations [11]. Furthermore, identifying the cell types of importance may accelerate the development of more targeted diagnostic strategies.

Cellular deconvolution using cfRNAs has been demonstrated to yield promising results in an increasing number of studies [6, 7, 12–15]. For instance, Vorperian et al. showed that hepatocyte signature scores are significantly higher in the blood of non-alcoholic steatohepatitis (NASH) and non-alcoholic fatty liver disease (NAFLD) patients compared with healthy control blood samples [18]. More widespread use of cellular deconvolution in liquid biopsy may result in it becoming an important component of the recently proposed "integrated liquid biopsy" framework [16], further increasing the sensitivity and specificity of available liquid biopsy biomarkers.

Liver cancer, predominantly represented by hepatocellular carcinoma (HCC) and intrahepatic cholangiocarcinoma (ICC), constitutes the second most lethal cancer type [16]. In 2020, liver cancer was responsible for more than 800,000 fatalities, and projections estimate a death toll of approximately 1,300,000 by the year 2040 [17]. The treatment of liver cancer, especially in advanced stages, is challenging, with the 5-year survival rate of HCC being just 18% [16]. Compounding the issue is the frequently delayed and late diagnosis of liver cancer, which can further diminish treatment efficacy [18] and the generally low accuracy of liver cancer diagnostic assays, particularly for early-stage detection [19–21]. Recently, liquid biopsy, including cfRNA biomarker discovery, has been proposed as a candidate to mitigate these issues [22–30] and hence, a number of publicly available cfRNA datasets have been generated. For these reasons, we decided to focus on investigating the cell type repertoire of blood cell-free transcriptome and its compositional changes in liver cancer.

Current cellular deconvolution methods typically rely on reference data that are advised to encompass all cell types present in the RNA mixture [9]. However, this strategy can make the analysis of non-hematopoietic cell types—particularly of rarer cell types—challenging, as hematopoietic cell types are the main contributor to the blood transcriptome [6, 31]. To address these challenges in blood cfRNA deconvolution and explore tissue or organ-specific cell-type proportion changes in the blood, we introduce a novel approach called targeted cellular deconvolution. Targeted cellular deconvolution involves deconvoluting cell-free transcriptomic data with the single-cell reference dataset of the tissue or organ of interest using the deconvolution algorithm Bisque [52]. This should ensure a comprehensive representation of cell types of interest and a more detailed examination of rarer cell types.

**Fig. 1:**
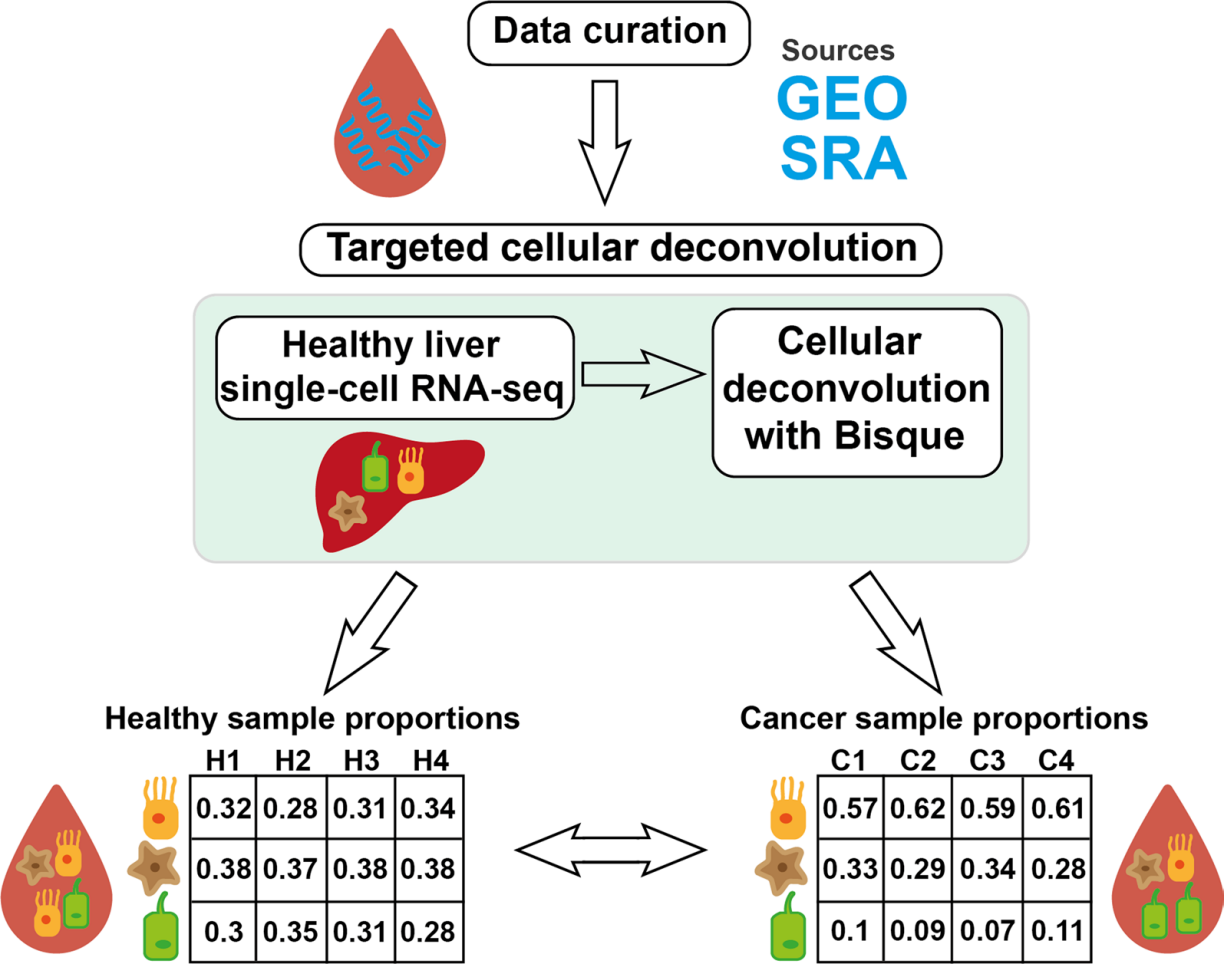
Workflow of targeted cellular deconvolution. After data curation from the Gene Expression Omnibus (GEO) and Sequence Read Archive (SRA) databases, the blood cell-free RNA datasets are deconvoluted with the Bisque algorithm using a healthy single-cell RNA liver dataset; the produced cell-type proportions are used to compare the healthy and cancer samples. H1–H4 represent the healthy donor samples, and C1–C4 represent the cancer samples

Here, by employing targeted cellular deconvolution, we analyzed over 350 blood cell-free RNA-seq samples gathered from four different studies [23–26], including over 200 liver cancer and healthy samples (Fig. 1). We detailed the observed cell-type proportion perturbations in liver cancer with emphasis on cell types displaying a high degree of discriminatory ability. Furthermore, by leveraging cell-type proportions, we constructed prognostic models, which were subsequently compared with models built using reported cell-free gene markers of liver cancer. Finally, we integrated cell type and gene-level information to build improved prognostic models for liver cancer diagnostics.

## Materials and methods

### Data processing

We used four publicly available cfRNA datasets containing blood RNA-seq samples from liver cancer patients and healthy donors, comprising 221 samples [23–26] (Table 1, Additional file 2: File S1). Additionally, the dataset from Chen et al. included 157 samples from patients of four types of solid cancers (Table 1) [25]. The dataset of Block et al. contained matched samples of extracellular vesicle and plasma RNA-seq from five liver cancer patients [23].

**Table 1:** Main characteristics of the cfRNA datasets used in the study

Dataset	HD	LC	STAD	LUAD	CRC	ESCA	Accession number	References
Roskams-Hieter et al. (2022)	30	28	NA	NA	NA	NA	SRP334205	[24]
Chen et al. (2022)	46	27	37	35	54	31	GSE174302	[25]
Zhu et al. (2021)	30	35	NA	NA	NA	NA	GSE142987	[26]
Block et al. (2022)	6	19	NA	NA	NA	NA	PRJNA907745	[23]

Metadata files were downloaded from Sequence Read Archive (SRA) database and supplemented by us with additional information from the corresponding studies. Datasets generated by Zhu et al. and Chen et al. provided gene count matrices, the generations of which are detailed in the corresponding studies and were downloaded from the Gene Expression Omnibus (GEO) database under the accession numbers GSE142987 and GSE174302, respectively (Table 1). The raw reads of datasets from Roskams-Hieter et al. and Block et al. were downloaded as raw FASTQ files from the SRA database using the tool “fasterq-dump” of the National Center for Biotechnology Information (NCBI) SRA-Tools (version 2.11.0) [32] under the accession numbers SRP334205 and PRJNA907745, respectively (Table 1). Subsequently, adapters were trimmed and ribosomal reads filtered by the BBDuk program of the BBMap suite of tools (version 38.93) [33]. Afterward, samples were mapped to the human genome (build GRCh38) using the default parameters of the aligner STAR (version 2.7.8a) [34]. Gene count matrices were generated by the function “featurecounts” of the Subread package of tools (version 2.0.1) [35], with the parameters “-p”, “-s 2” and “-M” using the human genome annotation of the GENCODE consortium (build GRCh38 release 38). The resulting matrices both comprised 60,708 genes and each contained 58 and 25 samples for Roskams-Hieter et al. and Block et al. datasets, respectively.

Ensembl gene IDs were converted to HGNC (Human Genome Organization Gene Nomenclature Committee) symbols using the R (version 4.1.2) [36] package biomaRt (version 2.50.3; Ensembl version 108) [37] to prepare the datasets for the subsequent cell-type deconvolution. As the dataset by Chen et al. already contained HGNC gene symbols, it was excluded from this step. The final Roskams-Hieter et al., Chen et al., Zhu et al. and Block et al. datasets contained 40,155, 13,4278, 68,696 and 40,155 genes, respectively. The dataset by Roskams-Hieter et al. was adjusted for batch effects using the function “Combat_seq” from the R package sva (version 3.42.0) [38], as this dataset was subsequently employed for model training. The samples were not further filtered or normalized as it has been shown that keeping samples in a linear scale ensures the best results during cell-type deconvolution [9] and the deconvolution algorithm performs its own internal gene filtering.

In order to generate principal component analysis (PCA) plots, samples were separately normalized with variance stabilizing transformation using the function “vst” from the R package DESeq2 (1.34.0) [39]. Plots were generated with the R package ggplot2 (version 3.4.1) [40].

### Cell-type deconvolution

Cell-type deconvolution was performed using the R package Bisque (version 1.0.5) [52] with a reference single-cell dataset. As the study focused on liver cancer, we used a liver-derived single-cell dataset hypothesized to predominantly capture liver-specific signals from the data. We selected the reference single-cell dataset in accordance with the guidelines set by the authors of the Bisque algorithm, which stipulate a minimum of three single-cell samples [52], and ensured that it featured robustly defined cell-type annotations. The reference single-cell dataset was generated by MacParland et al. from the livers of five healthy donors and contained cell type annotations for 8,444 cells [41]. The dataset containing log2CPM values and the corresponding annotation file were downloaded from the GEO database (accession number GSE115469). The single-cell reference data and the cell-free datasets were transformed into ExpressionSet class objects with the function “ExpressionSet” from the R package Biobase (version 2.54.0) [42]. To facilitate the cell-type deconvolution, the cell subtype annotations for hepatocytes, T cells, macrophages and liver sinusoidal endothelial cells (LSECs) were collapsed.

Finally, decomposition was carried out using the function “ReferenceBasedDecomposition” from the package Bisque with the parameter “use.overlap = FALSE” for each dataset. The Chen et al. dataset with the additional non-liver solid tumor samples was analyzed separately and was not used in the modeling steps.

### Statistical test computation

To test if hepatocyte proportions were greater in liver cancer samples compared to other samples, a one-sided, unpaired Wilcoxon test (Wilcoxon rank-sum test) was calculated using the deconvolution results of all samples with the function “wilcox_test” from the R package rstatix (version 0.7.2) [43]. To this end, the parameters “paired = FALSE,” “exact = TRUE” and “alternative = “greater” were used. For multiple comparisons, p-values were adjusted using the Benjamini–Hochberg method with the “adjust_pvalue” function and parameter “method = ”BH”” from the R package rstatix. Effect size (*r*) and corresponding confidence intervals were generated with the function “wilcox_effsize” using the parameters “alternative = ”greater”,” “paired = FALSE,” “nboot = 100″ and “ci = TRUE” from the R package rstatix.

To test if the hepatocyte proportions were greater in the plasma compared with extracellular vesicles (EVs) of five liver cancer patients, a one-sided, paired Wilcoxon test (Wilcoxon signed-rank test) was performed as previously described, with the only change being the parameter “paired = TRUE.” Effect size and corresponding confidence intervals were calculated as previously described, with the only change being “paired = TRUE.” The results were visualized with the R packages rstatix, ggpubr (version 0.5.0) [44] and ggplot2.

### Hepatocyte proportion-based classification

To analyze the feasibility of classifying liver cancer and healthy samples based on hepatocyte proportions, we tested 20 hepatocyte proportion cutoffs ranging from 0.2 to 0.4 in all cfRNA datasets—with samples above the cutoff classified as liver cancer (LC) patients and healthy donors (HD) if otherwise. Accuracy, sensitivity and specificity were computed at each cutoff with the function “confusionMatrix” from the R package caret (version 6.0–93) [45] and were used to generate a scatter plot using the R package ggplot2. A confusion matrix plot was generated at the cutoff with the highest classification accuracy with the function “evaluate” and a modified version of the function “plot_confusion_matrix” from the R package cvms (version 1.3.9.9000) [46].

## Model construction

### Random forest

We built random forest models to both determine the relative importance of cell types between biological conditions, sources of samples and to evaluate the diagnostic capabilities of various predictors. First, random forest models were built with each dataset using the cell-type proportions as input using the function “randomForest” with the parameter “importance = TRUE” from the R package randomForest (version 4.7–1.1) [47]. Afterward, the generated models were used as input for the function “varImpPlot” with the parameter “type = TRUE” from the R package randomForest, which calculates how much the model accuracy decreases without a certain predictor (feature). Finally, the results were visualized using the R package ggplot2.

To assess the performance of predictors, we trained a model with the Roskams-Hieter et al. dataset, chosen for its balanced structure and informative sample composition (Additional file 1: Fig. S1), using either the raw counts of gene markers reported by Roskams-Hieter et al. [24] and Chen et al. [25] (individually and combined) or integration of gene markers and four cell-type proportions (hepatocytes, LSECs, portal endothelial cells (PECs), cholangiocytes). The model was later tested on the remaining three cell-free datasets. First, through repeated cross-validation (five-fold; repeated five times), the optimal mtry hyperparameter value (controls number of sampled features) was determined for training a model with the Roskams-Hieter et al. dataset. To this end, the function “train” was used from the R package caret with the parameters “method = ”rf”,” “metric = ”ROC”” and “tunelenght = 10,″ which determined the mtry value with the highest achieved area under the receiver operator characteristic curve (AUROC/AUC). Then, the optimal mtry value was used to construct random forest models using the Roskams-Hieter et al. dataset as previously described (Additional file 1: Fig. S2).

A ROC curve was constructed with the out-of-bag (OOB) votes generated by the random forest algorithm using the function “roc” with the parameters “ci = TRUE” from the R package pROC (version 1.18.0) [48]. The ROC curve was utilized to determine the optimal cutoff for sample classification with the employment of the Youden *J* statistic. This was achieved with the function “coords” and the parameters “x = ”best”,” “best.method = ”Youden”” from the R package pROC.

Subsequently, the model was used to predict the probabilities of the class to which the samples belong from other cfRNA datasets with the “predict” function using the parameter “type = ”prob”” from the R package randomForest. The per dataset predicted probabilities were used as input to generate ROC curves as previously described. For the calculation of a total AUROC, all the predicted sample probabilities were used as input. Sample classification was performed using the cutoff value from the training dataset.

### Logistic regression

Equivalent to the random forest modeling, a logistic regression model with elastic net regularization was trained on the Roskams-Hieter et al. dataset with the cell-type proportion and later tested on three other cfRNA datasets. First, the optimal alpha and lambda hyperparameter values were determined, where alpha controls the extent of L1 and L2 regularizations and lambda determines the magnitude of the regularization. This was achieved by performing repeated cross-validation (five-fold; repeated five times) as previously described using the function “train” from the R package caret, where the parameters “method = ”glmnet”,” “metric = ”ROC,” “tunelenght = 10″ and “standardize = TRUE” were used. The resulting sample class probabilities were averaged across all the repeated cross-validations and used to assess the performance of the model. This was achieved by constructing a ROC curve as previously described (Additional file 1: Fig. S2).

The optimal hyperparameter values were used to train a model with the Roskams-Hieter et al. dataset with the function “glmnet” using the parameters “family = ”binomial”” and “standardize = TRUE” from the R package glmnet (version 4.1–6) [49]. To determine the optimal cutoff for sample classification, the model was tested on the Roskams-Hieter et al. dataset with the “predict” function and parameter “type = response” from the R package glmnet. The resulting probabilities were used as input for a ROC curve construction and the optimal cutoff was determined as previously described.

Finally, the generated model was tested on remaining cfRNA datasets using the function “predict” with the parameter “type = ”response”” from the R package glmnet. The predicted probabilities were used to generate ROC curves and calculate the total AUROC measurement as previously described. Sample classification was done using the cutoff value from the training dataset.

### Assessment of model performance

To assess the performance of the generated models, firstly, the AUROC was measured for each prediction with the function “calc_auc” from the R package plotROC (version 2.3.0) [50]. AUROC confidence intervals were obtained from the output of the “roc” function of the R package pROC. A confusion matrix plot was constructed for each model using the function “eval” and a modified version of the function “plot_confusion_matrix” from the R package cvms. Accuracy, sensitivity and specificity were calculated using the function “confusionMatrix” from the R package caret. Confidence intervals of accuracy were obtained from the output of the function “confusionMatrix” from the R package caret, while the confidence intervals of sensitivity and specificity were calculated with the function “epi_tests” with the default settings from the R package epiR (version 2.0.60) [51]. The results were visualized with the R packages ggplot2 and ggpubr.

To ascertain the influence of age on the accuracy of sample classification with the logistic regression model, we restricted our evaluation to male and female samples from healthy donors and liver cancer patients in the datasets generated by Roskams-Hieter et al. and Zhu et al., attributed to their comprehensive sample annotations. Visualization of sample metadata was conducted using the packages ggplot2 and ggpubr. Age-related differences in misclassified samples were statistically analyzed using an unpaired, two-sided Wilcoxon test as already described with the argument “alternative = ”t”” used in the “wilcox_test” and “wilcox_effsize” functions from the “rstatix” package.

We further explored the potential influence of the blood collection date on classification accuracy of the logistic regression model within the dataset from Block et al. as the dataset provided blood collection dates for liver cancer patient samples. First, liver cancer samples collected post-12–12-2010 were excluded, leading to the removal of six samples. Then, liver cancer samples obtained post-12–12-2016 were also omitted, resulting in four additional samples being excluded. Finally, in addition to testing the logistic regression model on the entirety of the Block et al. dataset, we also tested the model on the aforementioned filtered subsets as already described. Results were visualized using the ggplot2 and ggpubr packages as already described.

## Results

### Targeted cellular deconvolution validates hepatocyte importance in liver cancer blood transcriptomics

In order to investigate the liver cell type-specific contribution to the cell-free transcriptome in blood and its perturbations during carcinogenesis, we performed targeted cellular deconvolution on four cfRNA datasets containing 112 samples from healthy donors, 109 samples from liver cancer patients and 157 samples from colorectal cancer (CRC), stomach cancer (STAD), lung cancer (LUAD) and esophageal cancer (ESCA) patients (Table 1). The deconvolution was carried out with the Bisque algorithm and using a healthy single-cell liver reference dataset (Fig. 1). We chose Bisque for its displayed effectiveness, speed and robustness [52]. In particular, even in cases where the reference data may not fully represent all cell types, Bisque generates cell-type proportions that, although potentially not reflecting the absolute proportions within the mixture, still provide valuable insights into the biological condition of the mixed RNA-seq samples [52]. Lastly, Bisque has displayed effectiveness without needing the full repertoire of cell-type markers in the mixed RNA-seq samples [52]—an important consideration here, as dropouts cannot be ruled out for cfRNA sequencing.

**Fig. 2:**
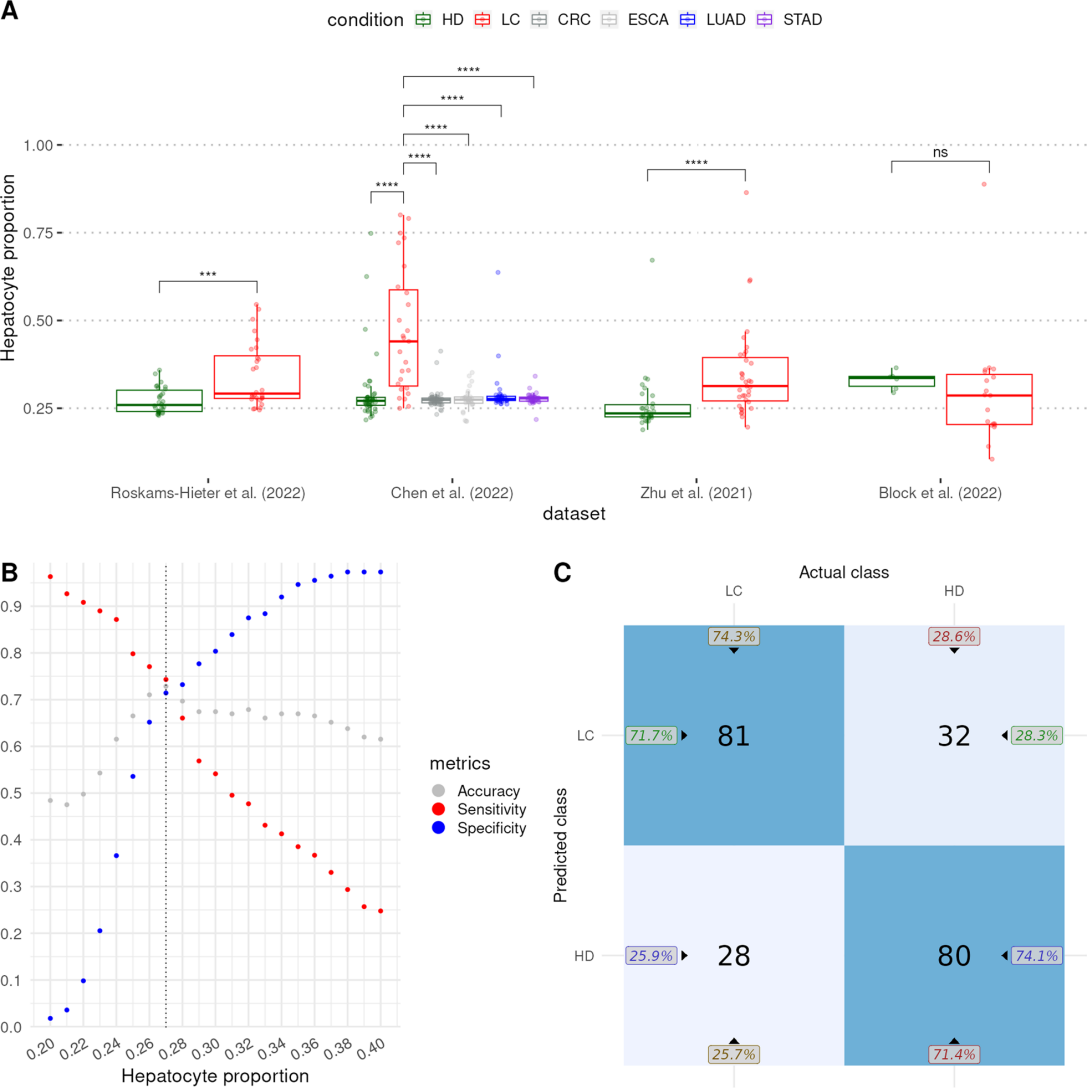
Analysis of hepatocyte proportions in cfRNA-seq datasets. **A** differences in hepatocyte proportions were analyzed with a one-sided Wilcoxon sum-rank test. *P* values were adjusted with the Benjamini–Hochberg procedure where appropriate. *ns* not significant, ***P < 10^–4^, ****P < 10^–5^. HD, healthy donor; LC, liver cancer; CRC, colorectal cancer; ESCA, esophageal cancer; LUAD, lung cancer; STAD, stomach cancer. **B** Hepatocyte proportion cutoff-based classification of HD and LC samples for proportions in the range of 0.2—0.4. Samples with a hepatocyte proportion higher than the tested proportion were classified as LC and HD if otherwise. The dotted vertical line indicates the most accurate hepatocyte proportion cutoff (0.27). **C** Confusion matrix of the results achieved after using the optimal hepatocyte proportion cutoff of 0.27. The left-hand top (71.7%) and right-hand bottom (74.1%) numbers represent the positive and negative predictive values, respectively. The numbers on the top (74.3%) and bottom (71.4%) represent the success rate of classifications

Owing to the displayed importance of hepatocytes in inferring liver health status, we further analyzed the hepatocyte proportions to assess the performance of the method. In three datasets, liver cancer patient samples had significantly larger hepatocyte proportions than the corresponding healthy donor samples (Fig. 2A). Only the smallest dataset—Block et al.—did not display any significant difference (Fig. 2A). In addition, in the Chen et al. dataset, we noticed significant differences in hepatocyte proportions between liver cancer samples and samples from other solid tumor samples, indicating that the abundance of hepatocyte RNA in the blood is directly related to the liver cancer of the patients (Fig. 2A). In the Roskams-Hieter et al. dataset, the differences in hepatocyte proportions had a moderate effect size (0.3 < *r* < 0.5), while in the Chen et al. and Zhu et al. datasets, the differences had a large effect size (*r* >  = 0.5) (Additional file 3: File S2).

Next, we tried to classify the healthy donor and liver cancer samples based on a hepatocyte proportion cutoff. We looked at cutoffs for hepatocyte proportions ranging from 0.2 to 0.4 and achieved the highest overall accuracy at a cutoff of 0.27 (Fig. 2B). Using the identified hepatocyte proportion cutoff of 0.27, in total 161 out of 221 samples were correctly classified with an accuracy of 72.85% (Fig. 2C, Additional file 4: File S3). The achieved positive predictive value (PPV) was 71.7% and the negative predictive value (NPV) was 74.1% (Fig. 2C). Although the overall accuracy was not very high, it is noteworthy that with the application of a simple hepatocyte proportion cutoff we were able to correctly classify most of the samples. This indicates the diagnostic potential of cell-type proportions in the blood.

### Targeted cellular deconvolution reveals dataset and biological source-dependent liver cell type proportion changes

**Fig. 3:**
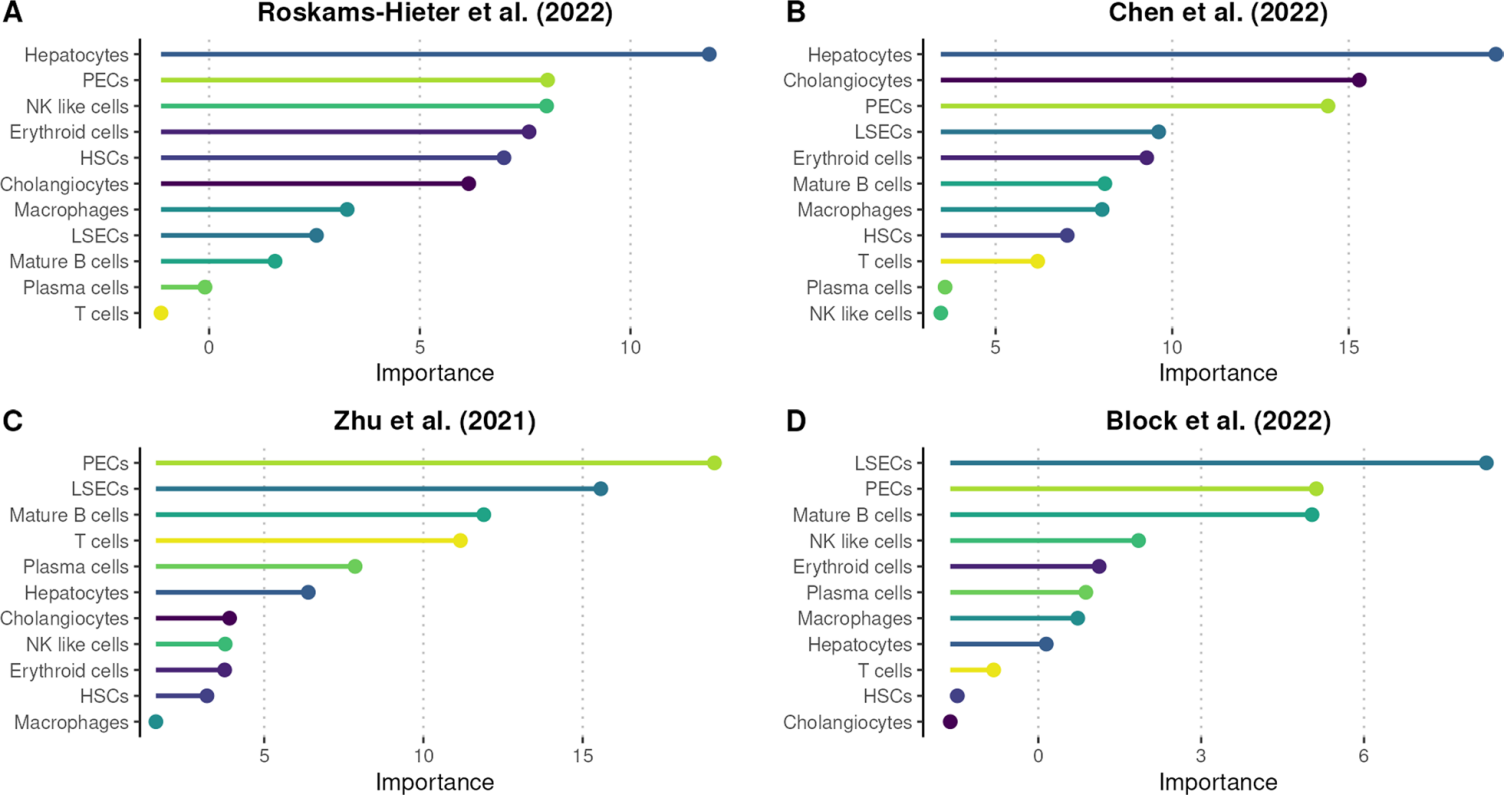
Cell type significance in differentiating healthy and cancerous samples. Random forest models were trained with Roskams-Hieter et al. (**A**), Chen et al. (**B**), Zhu et al. (**C**), Block et al. (**D**) datasets and the resulting Mean Decrease in Accuracy (MDA) measurement was used to order the cell types in descending order of importance for the classification of HD and LC samples in each dataset. HD, healthy donor; LC, liver cancer; PECs, portal endothelial cells; NK, natural killer, HSCs, hepatic stellate cells; LSECs, liver sinusoidal endothelial cells

Building on the encouraging results displayed by analyzing hepatocyte proportions, we decided to broaden the scope of our analysis and investigate other liver cell types as well. To that end, we used the cell-type proportions to analyze their relative importance for separating healthy and liver cancer samples per dataset. A random forest model was fitted with each dataset and the resulting Mean Decrease in Accuracy (MDA) measure was used to rank the features (Fig. 3). In the Roskams-Hieter et al. (Fig. 3A) and Chen et al. (Fig. 3B) datasets, hepatocytes displayed the highest importance. In comparison, the datasets from Zhu et al. (Fig. 3C) and Block et al. (Fig. 3D) highlight the importance of endothelial cell types such as liver sinusoidal endothelial cells (LSECs) or portal endothelial cells (PECs). Interestingly, the high importance of PECs was consistent across all datasets examined (Fig. 3A–D). Also noteworthy, cholangiocytes were the second-highest-importance cell type in the Chen et al. dataset, considering the presence of some ICC-derived samples in the dataset (Additional file 2: File S1).

Next, we investigated the difference in cell type proportions between five plasma and extracellular vesicle (EV) samples from the same liver cancer patients in the Block et al. dataset (Additional file 1: Fig. S3). Random forest models built with the datasets showed erythroid cells and hepatocytes having the highest importance in differentiating plasma and EV samples (Additional file 1: Fig. S3A). In all five cases, plasma samples had higher hepatocyte proportions than the corresponding EV samples (Additional file 1: Fig. S3B, Additional file 3: File S2), which agrees with a previously made observation [53].

### Targeted cellular deconvolution accurately and robustly classifies cancer samples

In order to assess the feasibility of diagnostic modeling using the cellular deconvolution output, we used the cell-type proportions as input for logistic regression and random forest models. Since the random forest modeling yielded comparatively less accurate results, we decided to continue with a logistic model with elastic net regularization for classification based on targeted cellular deconvolution results. This strategy helps to combat possible overfitting by penalizing and excluding less informative features. A model was trained using the dataset by Roskams-Hieter et al. (Additional file 1: Fig. S3, Additional file 4: File S3) and was subsequently tested on the remaining three cfRNA datasets comprising in total of 82 samples from non-cancer donors and 81 samples from liver cancer patients. The dataset by Roskams-Hieter et al. was chosen for its balanced nature, a high number of liver cancer samples, and a potentially more challenging and informative sample composition (Additional file 1: Fig. S1). The results of the model testing showed a total AUROC of 0.85 and stable AUROC measurements across the individual datasets (Additional file 1: Fig. S4A). In total, 128 samples out of 163 were correctly classified with a positive predictive value (PPV) of 80.3% and a negative predictive value (NPV) of 77% (Additional file 1: Fig. S5A).

To study the influence of potential confounding factors on the efficacy of the logistic regression model, we initially examined the age distribution within the male and female samples from the Roskams-Hieter et al. and Zhu et al. datasets (Additional file 1: Fig. S6A) with a particular focus on the misclassified samples (Additional file 1: Fig S6B). Our findings revealed that among all comparisons only two had statistically significant (*P* = 0.03) age disparities: between female liver cancer samples and their male counterparts, and between female healthy donors and female liver cancer samples (Additional file 3: File S2). No statistically significant age variations were observed among the misclassified male and female samples or between the misclassified and the entire sample set (Additional file 3: File S2). This suggests that despite certain biases present in the datasets from Roskams-Hieter et al. and Zhu et al., the classification accuracy of the model remained unaffected.

Additionally, we considered the effect of blood collection dates on the accuracy of the model within the Block et al. dataset. After excluding liver cancer samples collected post-2010, the AUROC of the model increased to 0.91 from an initial 0.79, which was observed on the complete dataset (Additional file 1: Fig. S7). A further exclusion of samples collected post-2016, elevated the AUROC to 0.98 (Additional file 1: Fig. S7). These enhancements suggest that prolonged storage of blood samples can negatively impact the targeted cellular deconvolution model performance. This deterioration in performance could stem from storage conditions leading to decreased sample quality.

Next, we compared the performance of the deconvolution-based diagnostic model with models based on the cell-free gene markers reported by Roskams-Hieter et al. (10 markers), Chen et al. (5 markers) and a combination of both (15 markers). Since the logistic regression modeling yielded comparatively less accurate results, we decided to continue with a random forest model. A random forest model was trained using the Roskams-Hieter et al. dataset using either the marker sets separately or combined (Additional file 1: Fig. S2, Additional file 4: File S3). The model testing results showed a total AUROC of 0.81, 0.84 and 0.82 for Roskams-Hieter et al., Chen et al. and combined models with Block et al. dataset predictions displaying relatively low AUROC in all three cases (Additional file 1: Figure S4B-D). In total, Roskams-Hieter et al. and Chen et al. gene marker models correctly classified 122 samples, while the combined model—126 (Additional file 1: Fig. S5B-D). The displayed PPV and NPV for Roskams-Hieter et al., Chen et al. and combined gene markers were 81.2%, 76.3%, 86.7% and 70.7%, 73.6%, 71.8%, respectively (Additional file 1: Fig. S5B-D). Overall, these results demonstrate that models built with the results of cellular deconvolution of cfRNA are yielding classification results that are comparable to established markers, thus confirming the validity of this approach.

### Integration of cell-type proportions into gene-marker models leads to improved performance

**Fig. 4:**
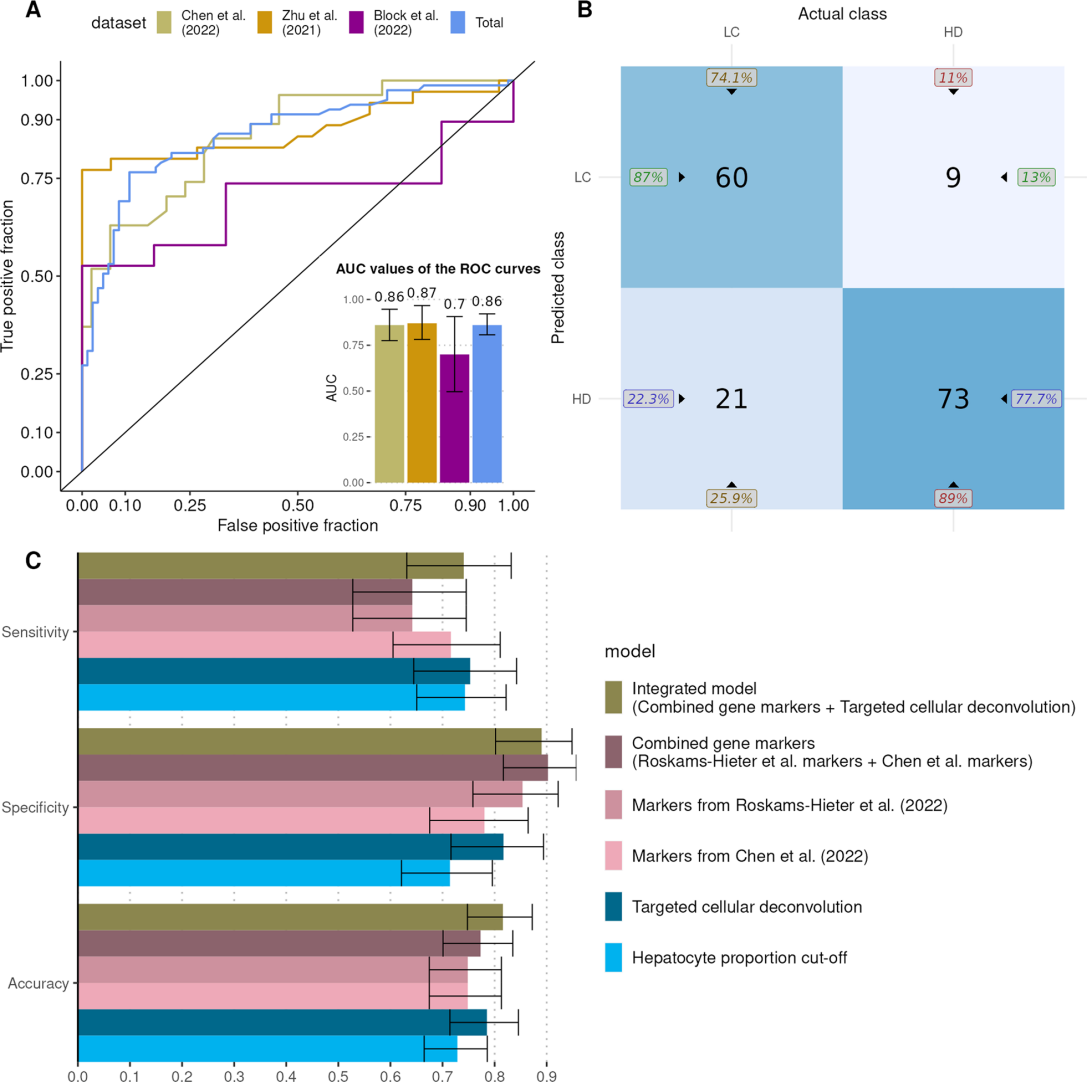
Performance assessment of the integrated model. Proportions of hepatocytes, cholangiocytes, liver sinusoidal and portal endothelial cells were integrated with gene markers described in the literature. The resulting integrated model was trained with Roskams-Hieter et al. (2022) dataset and tested on the remaining datasets. The results were assessed with receiver operator characteristic (ROC) curves and area under ROC curves (AUC) values (**A**), a confusion matrix (**B**) and were compared with the performance of other models (**C**). The error bars depicted in the figure represent the 95% confidence intervals

Next, we aimed to test if integrating cell-type proportions into gene marker-based diagnostic models would improve classification results. In order to achieve that, we added four cell-type proportions (hepatocytes, LSECs, PECs, cholangiocytes) that previously displayed high importance in differentiating biological conditions to the combined gene marker random forest model. As before, the integrated model was trained with the Roskams-Hieter et al. dataset (Additional file 1: Fig. S2) and tested on three other cfRNA datasets (Additional file 4: File S3). Compared with the targeted cellular deconvolution and individual gene marker-based models, after model testing the integrated model achieved a higher total AUROC (0.86) (Fig. 4A) and higher PPV and NPV—87% and 77.7%, respectively (Fig. 4B). The integrated model correctly classified 133 samples, thereby outperforming the other models (Fig. 4B).

To analyze the performance of the models further, we also examined the accuracy, specificity and sensitivity that the models achieved (Fig. 4C). For these comparisons, we also included the hepatocyte proportion cutoff-based classification that we described in Fig. 2C with the exclusion of the Roskams-Hieter et al. dataset results. The integrated model demonstrated the highest accuracy (0.816) and matched the specificity (0.89) that was obtained from the combined gene marker model (0.902), which exhibited the highest specificity among all models (Fig. 4C). The targeted cellular deconvolution-based model achieved the highest sensitivity (0.753), which is comparable to the integrated model (0.74) (Fig. 4C). Notably, the sensitivity attained by the integrated model surpassed that of the most sensitive gene marker model (Chen et al.), which had a sensitivity of 0.716 (Fig. 4C). These results support the notion that the synthesis of cell-type proportions and gene marker data can result in more accurate diagnostic models.

## Discussion

Liver cancer diagnosis remains a challenge despite numerous advancements made toward elucidating its progression and early presentation. Liquid biopsy holds great promise in providing a readily available and potentially more robust diagnostic approach but is hindered by the fragmented nature of existing research and the absence of comprehensive efforts to synthesize current findings. Additionally, as liquid biopsy is still a developing field, it still holds substantial untapped potential. One of the still poorly explored aspects of liquid biopsy and specifically blood cell-free transcriptome, is the degree of the contribution of various cell types. Here, we applied a novel deconvolution approach—targeted cellular deconvolution—to over 350 blood cfRNA samples that using the Bisque algorithm, deconvolutes the RNA mixture utilizing the tissue or organ of interest (i.e., liver).

The targeted cellular deconvolution provided a snapshot of the contribution of liver cell types to the blood cell-free transcriptome. By extrapolating the cell-type proportions, liver-targeted cellular deconvolution also provided the changes in liver cell-type proportions during cancerogenesis. As for the liver [54], the cell type with the largest contribution is the hepatocytes. We found that hepatocyte proportions were increased significantly in most datasets in liver cancer patients compared with not only healthy but also other solid tumor blood samples. This displays not only the perturbations of hepatocytes during cancerogenesis but also their specificity to liver cancer. Hepatocyte cell death is assumed to be an important step of HCC progression [55, 56] and may explain the increase of hepatocyte signal in the blood of liver cancer patients, the vast majority of whom were diagnosed with HCC (Additional file 2: File S1). With this in mind, we tried to separate healthy and liver cancer samples using hepatocyte proportions. At a cutoff of 0.27, we were able to correctly classify most of the samples with almost equal accuracy in detecting healthy and liver cancer samples. It is especially noteworthy taking into account the varied origins of the samples, the preponderance of early-stage liver cancer samples and samples with blood alpha-fetoprotein (AFP) levels below the commonly used diagnostic threshold of 400 ng/ml [20] (Additional file 2: File S1).

We further investigated the importance of other liver cell types in classification. Hence, we fitted random forest models with each dataset and, through the MDA measurement, ordered the cell types by importance in each dataset. While the high importance displayed by hepatocytes in two datasets was expected, it was interesting to note that in two other datasets, other cell types, notably LSECs and PECs, were more important in classification. PECs were also highly important in all four datasets, further strengthening the argument for an increased focus on the liver endothelial cells. While LSECs have recently received attention for their role in liver cancer cancerogenesis [57] and potential in liquid biopsy research [58], PECs still remain relatively poorly characterized and their role in liver cancer is not fully elucidated. However, as in the case of LSECs [59], their proximity to blood vessels [41] makes PECs a possible source of information on the liver condition in blood. Finally, cholangiocytes, which were relatively low in importance across three datasets, were the second highest important cell type in the Chen et al. generated dataset. The Chen et al. dataset contained some ICC patient-derived samples that could explain the displayed importance of cholangiocytes [60]. This indicates that cholangiocyte contribution to blood cell-free transcriptome warrants further investigation, given that ICC is the second most widespread primary liver cancer type.

A recent consideration in the field of liquid biopsy has been the source of the cell-free RNAs in blood, with the main choices being between plasma and EVs [12, 23, 53]. Our analysis of the matched plasma and EV samples from five liver cancer patients in the Block et al. dataset showed the high importance of erythroid cells and hepatocytes in separating these samples. Erythrocyte proportions have been reported to be increased in EVs compared with plasma [12], which is supported by our results. Additionally, in matched HCC-derived samples we found significantly higher proportions of hepatocytes in plasma compared with EVs, which is consistent with a previous observation in HCC patients of higher expression of circulating liver-derived transcripts in plasma compared with EVs [53]. Our results lead us to believe that EV isolation may be unnecessary for liver cancer diagnosis, especially considering that EV isolation is very labor intensive.

We also endeavored to improve upon the results displayed by the hepatocyte proportion cutoff. Since we noticed that hepatocytes did not have a strong discriminatory ability in the Block et al. dataset and other cell types also displayed high importance during classification, we sought to incorporate the proportions of other liver cell types as well into developing more robust diagnostic models. Logistic regression with an elastic net regularization model built with all the cell-type proportions of Roskams-Hieter et al. datasets as input showed robust results across all three tested datasets. Compared with the hepatocyte proportion cutoff-based classification, the overall accuracy was improved, proving further the importance of other, non-hepatocyte cell types as well. To assess the performance of targeted cellular deconvolution models, we concurrently analyzed some of the reported cell-free gene marker sets for liver cancer diagnosis. The gene marker sets were modeled both individually and in combination. Random forest models built using gene marker expression in the Roskams-Hieter et al. dataset displayed comparable accuracy to the deconvolution-based model, with the latter slightly outperforming the former. While the cellular deconvolution model displayed higher sensitivity, the gene marker models (especially the combined model) outperformed it in terms of specificity. These results not only show the validity of both methods but also underline the remarkable similarity and stability of blood cfRNA samples generated in different environments and drawn from patients with different etiologies (e.g., hepatitis B, hepatitis C, hepatic steatosis) (Additional file 2: File S1).

In light of the strengths of each diagnostic model and the enhanced performance of the combined gene marker model, we decided to integrate some of the cellular deconvolution results into the combined gene markers models. Based on previous results, we decided to integrate the proportions of hepatocytes, cholangiocytes, PECs and LSECs into the combined gene marker model. The new, integrated model displayed the highest overall accuracy among all models and closely matched the sensitivity and specificity of the deconvolution and gene marker models. The enhanced performance of the integrated model thus facilitates more comprehensive modeling of liquid biopsy data, incorporating not only gene marker expression but also additional data, such as cell-type proportions. We expect that integrated models will exhibit improved performance as they will incorporate more and varied types of liquid biopsy data. Particularly with the concern of relatively low sensitivity displayed by prospective liquid biopsy assays, the incorporation of cell-type proportions yielded by targeted cellular deconvolution can mitigate that issue to a degree.

Potential confounding factors remain a major issue for the clinical adoption of liquid biopsy. A comprehensive exploration of potential sources of variation in the blood cell-free transcriptome can mitigate these concerns. While our analysis showed one of the major confounders in liquid biopsy—age [61, 62]—to have no discernible effect on the efficacy of the targeted cellular deconvolution model either for male or female samples, we identified sample generation date to be of vital importance. Although sometimes unavoidable, extended storage of blood samples, especially in improper conditions, should be avoided whenever possible for optimal outcomes. Yet, further exploration is needed to identify other unknown confounders and possibly mitigate their adverse effects.

## Conclusions

In conclusion, in this study, we showed the viability of liquid biopsy studies that are translatable across different conditions. Furthermore, we highlighted the potential of targeted cellular deconvolution and deconvolution in general for blood cell-free transcriptomic studies, which can improve cfRNA characterization and assist in the development of enhanced diagnostic assays.

In the future, we envision the application of targeted cellular deconvolution to other conditions as well and the expansion of the data generated by us through the deeper analysis of, for example, liver cirrhosis-derived samples. The increase of assay accuracy with the addition of cell-type proportion data to other liquid biopsy biomarkers in the framework of “integrated liquid biopsy” can facilitate its clinical adoption. Finally, as new liquid biopsy datasets are being continuously generated, the need for meta-analyses, comparison and integration of diverse and extensive information will continue to grow and we expect more gene markers will be discovered. We believe that the strategies outlined in this study will contribute to these efforts and expedite the clinical adoption of liquid biopsy diagnostic assays.

### Supplementary Information

**Additional file 1**. **Figure S1:** Principal component analysis (PCA) plot. Plots were generated using variance stabilized counts prior to batch correction for Roskams-Hieter *et al*. (**A**), Chen *et al*. (**B**), Zhu *et al*. (**C**) and Block *et al*. (**D**) datasets. HD, healthy donor; LC, liver cancer. **Figure S2:** Performance of model training with Roskams-Hieter *et al*. (2022) dataset. The results are represented with receiver operator characteristic (ROC) curves and area under ROC curves (AUC) values. **Figure S3:** Differences in cellular sources of cfRNA between matched plasma and extracellular vesicle (EV) samples in the Block *et al*. (2022) dataset. (**A**) Importance of cell types in the classification of plasma and EV samples as measured by the Mean Decrease in Accuracy (MDA) value from the random forest model. Cell types were ordered in descending order of MDA. (**B**) Hepatocyte proportion differences between matched plasma and EV samples from each patient. **Figure S4:** Performance of model testing on each dataset represented with receiver operator characteristic (ROC) curves and area under ROC (AUC) values. The targeted cellular deconvolution (**A**), Roskams-Hieter *et al*. gene markers (**B**), Chen *et al*. gene markers (**C**) and combined gene markers (**D**) models were trained with the Roskams-Hieter *et al*. (2022) dataset and tested on the remaining datasets. The results were assessed with receiver operator characteristic (ROC) curves and area under ROC curves (AUC) values . The error bars depicted in the figure represent the 95% confidence intervals. **Figure S5:** Confusion matrices of model testing. Confusion matrices for the targeted cellular deconvolution (**A**), Roskams-Hieter *et al*. gene markers (**B**), Chen *et al*. gene markers (**C**) and combined gene markers (**D**) model testing. The left-hand top and right-hand bottom numbers represent the positive and negative predictive values, respectively. The numbers on the top and bottom represent the success rate of classifications. **Figure S6:** Age distribution in Roskams-Hieter *et al*. and Zhu *et al*. datasets. Age distribution of female and male healthy donor (HD) and liver cancer (LC) samples in Roskams-Hieter *et al*. and Zhu *et al*. datasets (only these datasets contained comprehensive per sample annotations) (**A**), all samples (**B**), samples misclassified by the targeted cellular deconvolution model. **Figure S7:** Influence of sample collection date on model performance. Performance of targeted cellular deconvolution model on Block *et al*. dataset (only this dataset contained bleed date information): full, after removing liver cancer samples generated after 2010 and after further removing liver cancer samples generated after 2016. The results are represented with receiver operator characteristic (ROC) curves and area under ROC curves (AUC) values.

**Additional file 2.** Detailed overview of liver cancer patient characteristics across datasets.

**Additional file 3.** Results of statistical analyses.

**Additional file 4.** Classification and modeling results.

## Data Availability

The public datasets analyzed during the current study are available in the NCBI GEO repository under accession numbers GSE174302 (https://www.ncbi.nlm.nih.gov/geo/query/acc.cgi?acc=GSE174302), GSE142987 (https://www.ncbi.nlm.nih.gov/geo/query/acc.cgi?acc=GSE142987), GSE115469 (https://www.ncbi.nlm.nih.gov/geo/query/acc.cgi?acc=GSE115469) and NCBI SRA repository under accession numbers SRP334205 (https://www.ncbi.nlm.nih.gov/sra?term=SRP334205) and PRJNA907745 (https://www.ncbi.nlm.nih.gov/bioproject/PRJNA907745). The computational code used in this study is available at GitHub: https://github.com/aramsafrast/cfdeconv.
