# Supplementary material for: Decoding cell-type contributions to the cfRNA transcriptomic landscape of liver cancer
Source: Hum Genomics. 2023 Oct 5;17:90. doi: 10.1186/s40246-023-00537-w (PMC10552294; doi:10.1186/s40246-023-00537-w)
Supplement: Supplementary file 1 — Additional file 1. Figure S1: Principal component analysis (PCA) plot. Plots were generated using variance stabilized counts prior to batch correction for Roskams-Hieter et al. (A), Chen et al. (B), Zhu et al. (C) and Block et al. (D) datasets. HD, healthy donor; LC, liver cancer. Figure S2: Performance of model training with Roskams-Hieter et al. (2022) dataset. The results are represented with receiver operator characteristic (ROC) curves and area under ROC curves (AUC) values. Figure S3: Differences in cellular sources of cfRNA between matched plasma and extracellular vesicle (EV) samples in the Block et al. (2022) dataset. (A) Importance of cell types in the classification of plasma and EV samples as measured by the Mean Decrease in Accuracy (MDA) value from the random forest model. Cell types were ordered in descending order of MDA. (B) Hepatocyte proportion differences between matched plasma and EV samples from each patient. Figure S4: Performance of model testing on each dataset represented with receiver operator characteristic (ROC) curves and area under ROC (AUC) values. The targeted cellular deconvolution (A), Roskams-Hieter et al. gene markers (B), Chen et al. gene markers (C) and combined gene markers (D) models were trained with the Roskams-Hieter et al. (2022) dataset and tested on the remaining datasets. The results were assessed with receiver operator characteristic (ROC) curves and area under ROC curves (AUC) values . The error bars depicted in the figure represent the 95% confidence intervals. Figure S5: Confusion matrices of model testing. Confusion matrices for the targeted cellular deconvolution (A), Roskams-Hieter et al. gene markers (B), Chen et al. gene markers (C) and combined gene markers (D) model testing. The left-hand top and right-hand bottom numbers represent the positive and negative predictive values, respectively. The numbers on the top and bottom represent the success rate of classifications. Figure S6: Age distribution in Roskams- [file 40246_2023_537_MOESM1_ESM.docx]

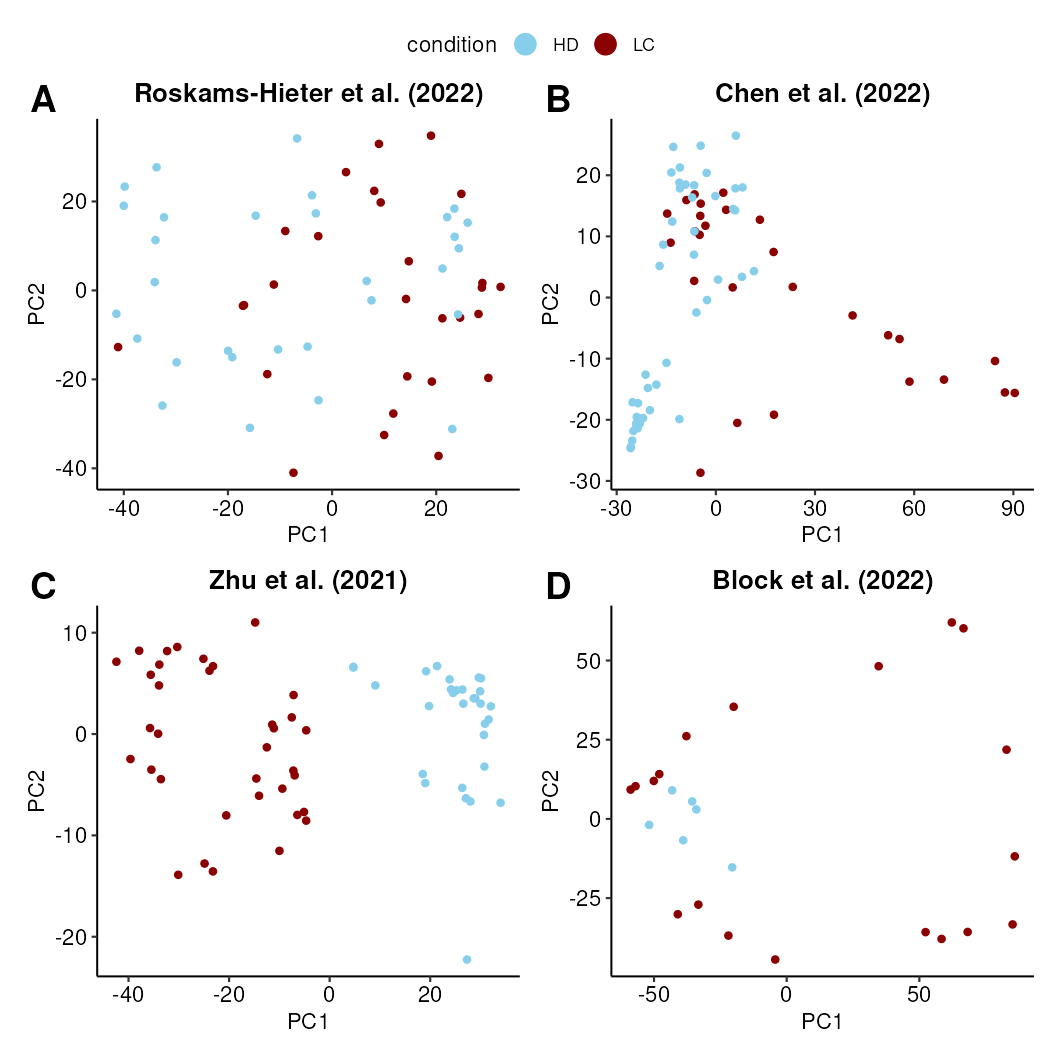


**Suppl. Figure 1**: **Principal component analysis (PCA) plot.** Plots were generated using variance stabilized counts prior to batch correction for Roskams-Hieter *et al*. (**A**), Chen *et al*. (**B**), Zhu *et al*. (**C**) and Block *et al*. (**D**) datasets. HD, healthy donor; LC, liver cancer.


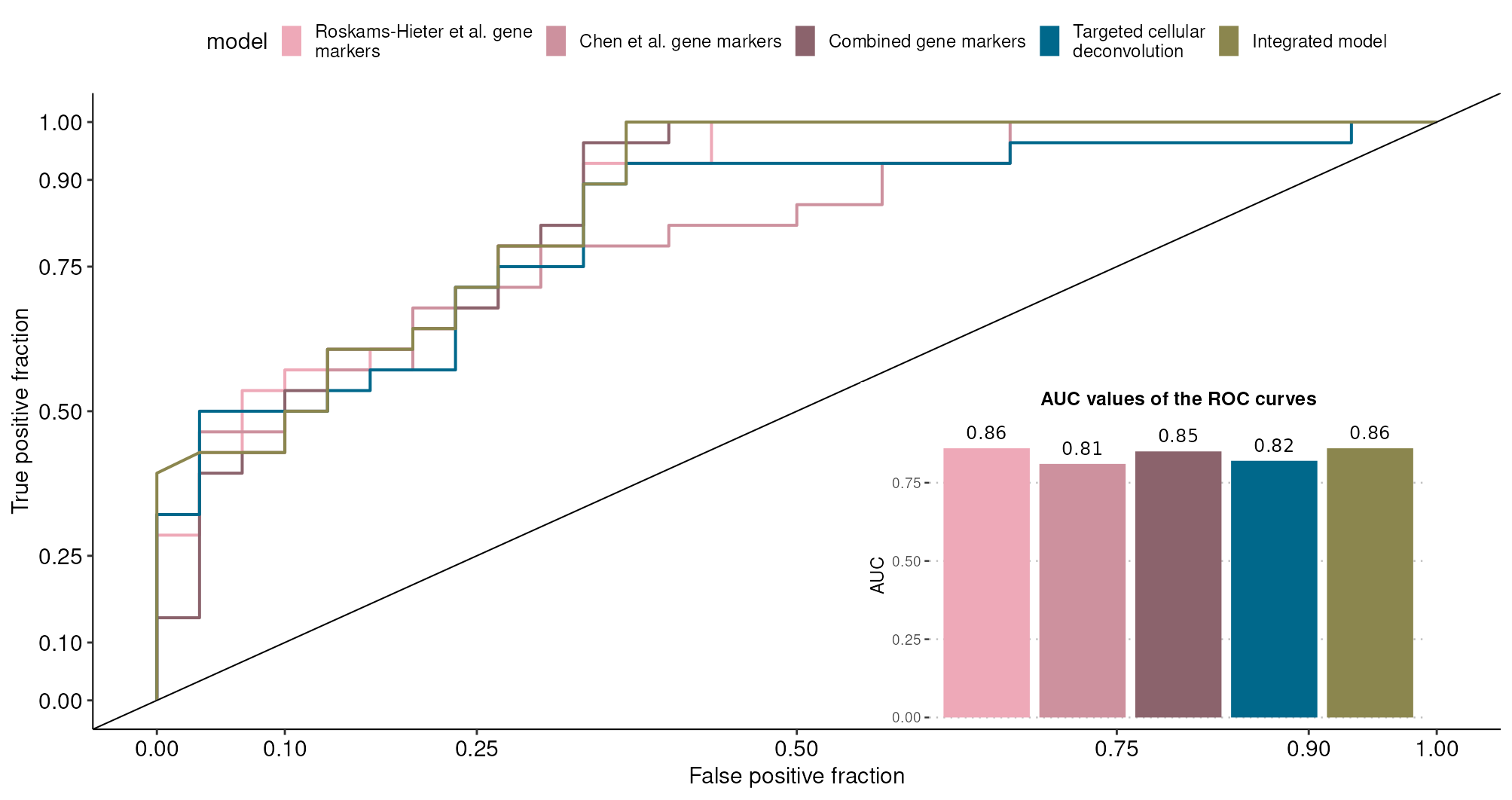


**Suppl. Figure 2**: **Performance of model training with Roskams-Hieter *et al*. (2022) dataset.** The results are represented with receiver operator characteristic (ROC) curves and area under ROC curves (AUC) values.


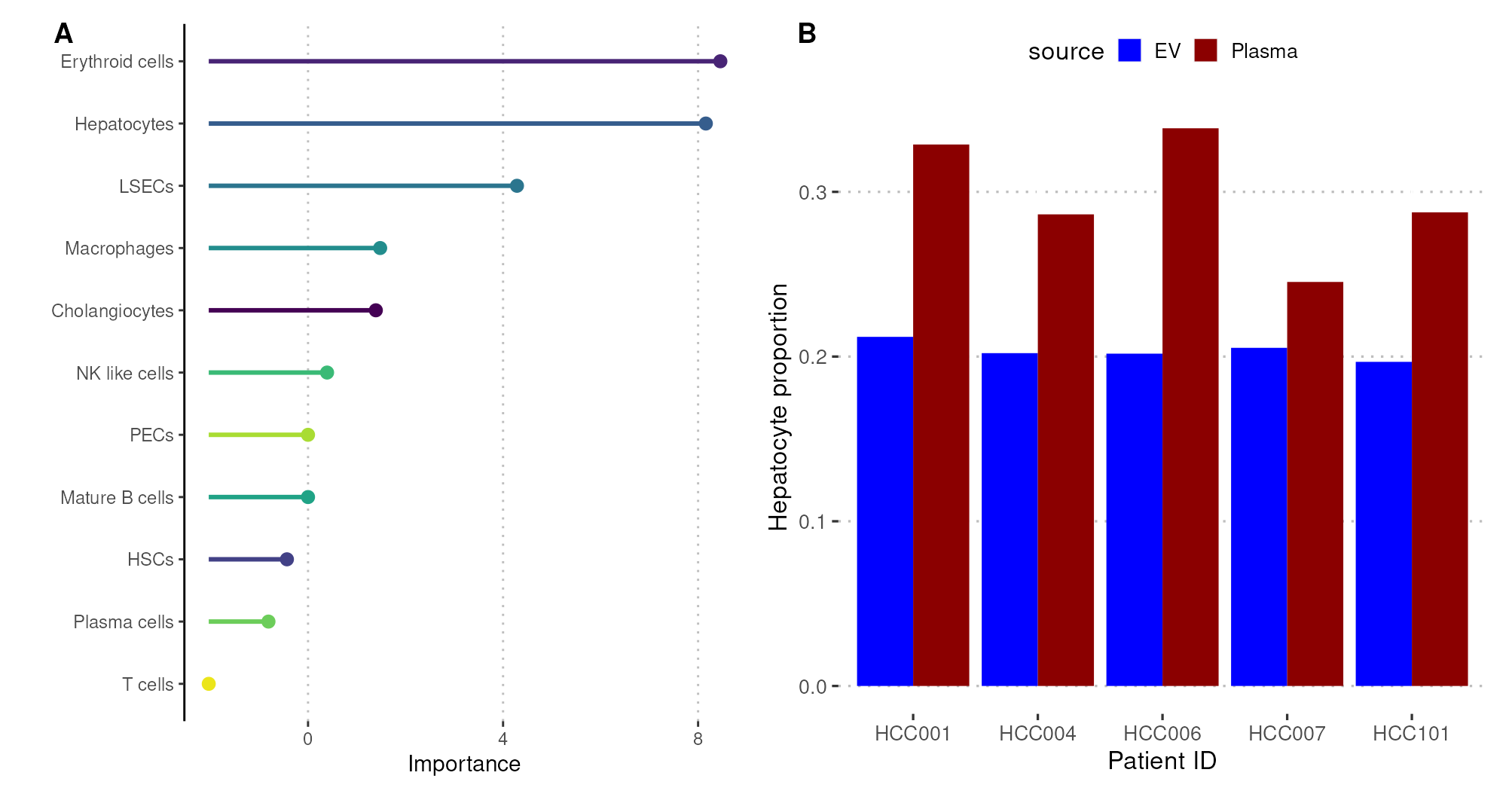


**Suppl. Figure 3**: **Differences in cellular sources of cfRNA between matched plasma and extracellular vesicle (EV) samples in the Block *et al*. (2022) dataset**. (**A**) Importance of cell types in the classification of plasma and EV samples as measured by the Mean Decrease in Accuracy (MDA) value from the random forest model. Cell types were ordered in descending order of MDA. (**B**) Hepatocyte proportion differences between matched plasma and EV samples from each patient.


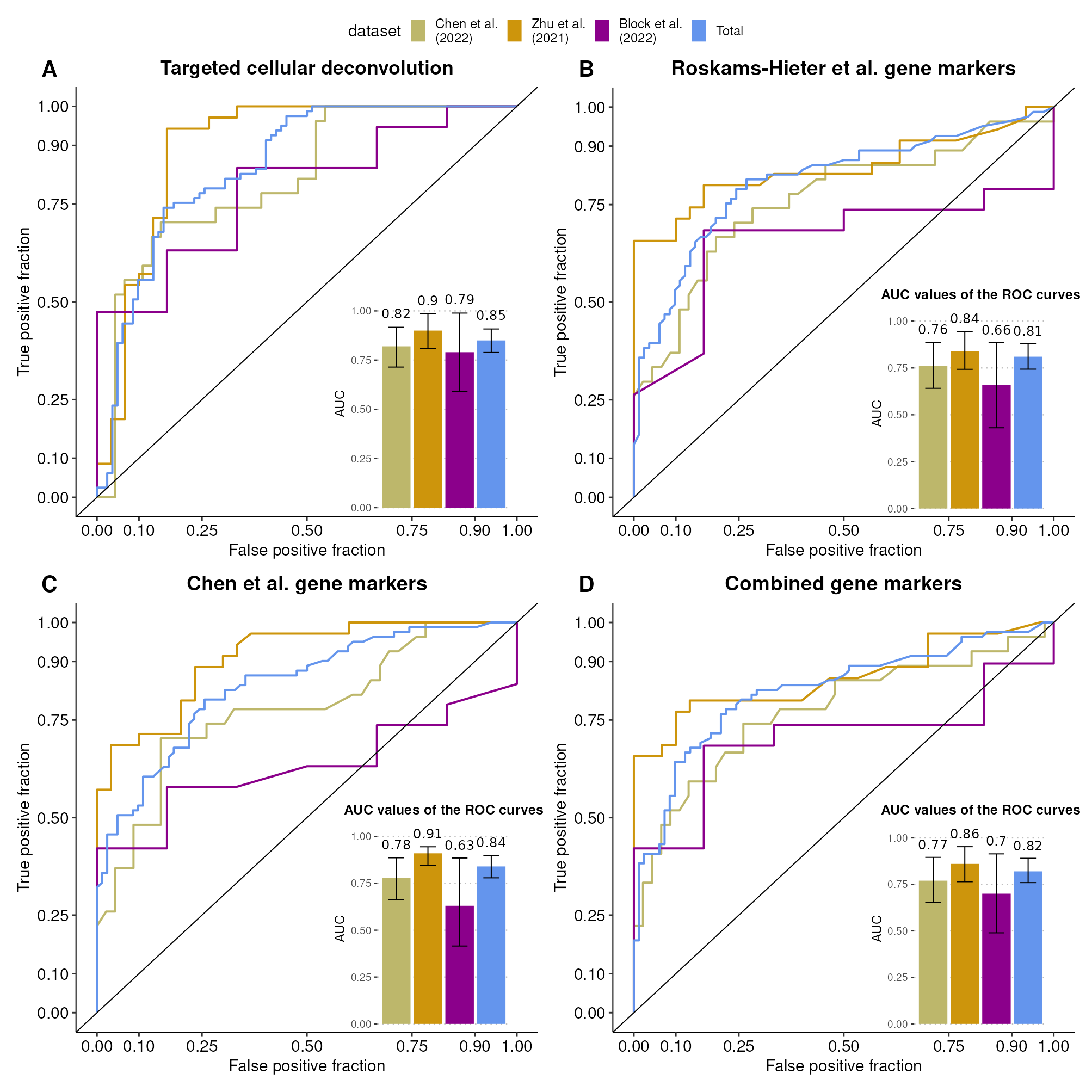


**Suppl. Figure 4**: **Performance of model testing on each dataset represented with receiver operator characteristic (ROC) curves and area under ROC (AUC) values**. The targeted cellular deconvolution (**A**), Roskams-Hieter *et al*. gene markers (**B**), Chen *et al*. gene markers (**C**) and combined gene markers (**D**) models were trained with the Roskams-Hieter *et al*. (2022) dataset and tested on the remaining datasets. The results were assessed with receiver operator characteristic (ROC) curves and area under ROC curves (AUC) values . The error bars depicted in the figure represent the 95% confidence intervals.


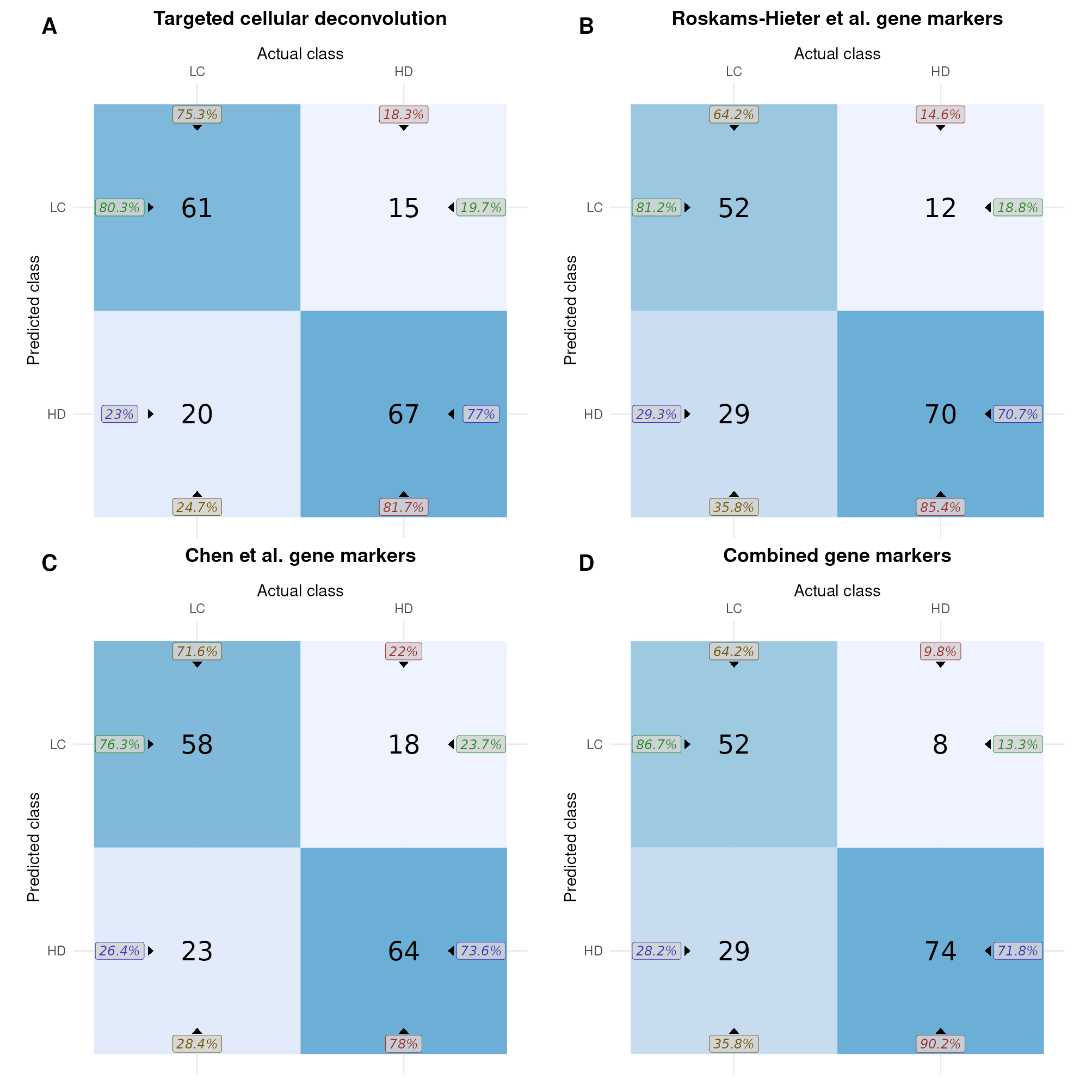


**Suppl. Figure 5**: **Confusion matrices of model testing**. Confusion matrices for the targeted cellular deconvolution (**A**), Roskams-Hieter *et al*. gene markers (**B**), Chen *et al*. gene markers (**C**) and combined gene markers (**D**) model testing. The left-hand top and right-hand bottom numbers represent the positive and negative predictive values, respectively. The numbers on the top and bottom represent the success rate of classifications.

**Suppl. Figure 6**: **Age distribution in Roskams-Hieter *et al*. and Zhu *et al*. datasets**. Age distribution of female and male healthy donor (HD) and liver cancer (LC) samples in Roskams-Hieter *et al*. and Zhu *et al.* datasets (only these datasets contained comprehensive per sample annotations) (**A**), all samples (**B**), samples misclassified by the targeted cellular deconvolution model.
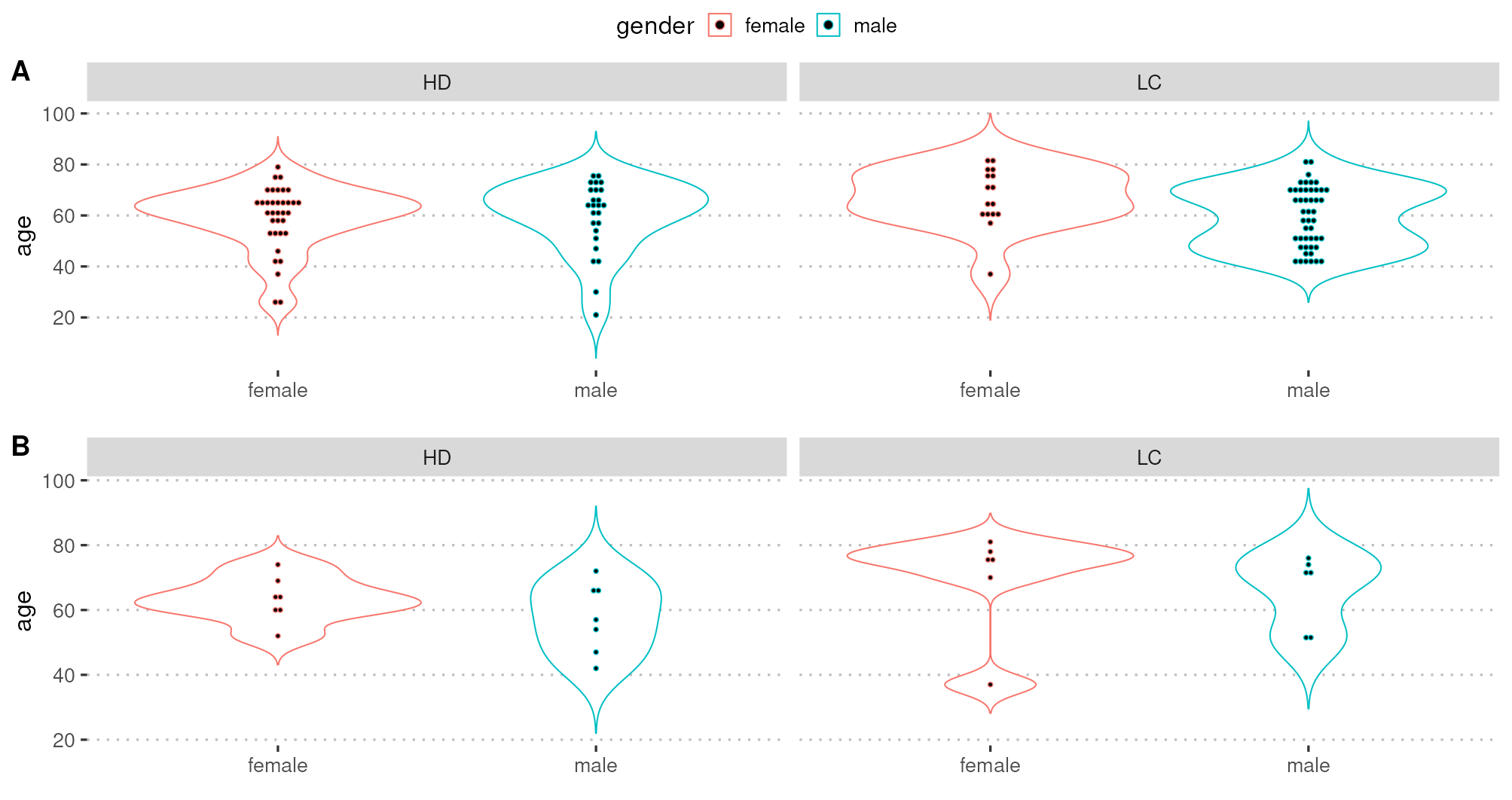


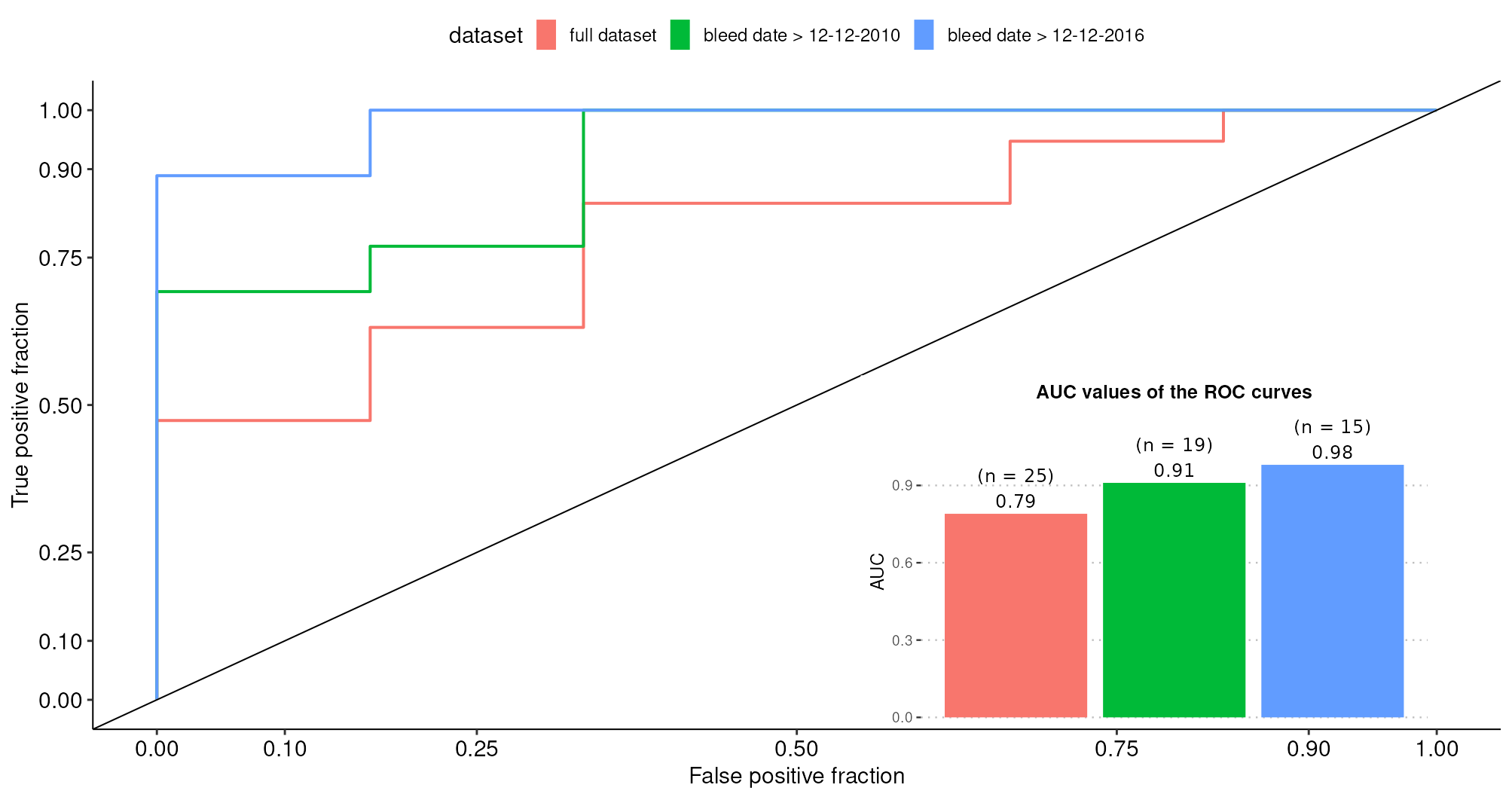
**Suppl. Figure 7**: **Influence of sample collection date on model performance**. Performance of targeted cellular deconvolution model on Block et al. dataset (only this dataset contained bleed date information): full, after removing liver cancer samples generated after 2010 and after further removing liver cancer samples generated after 2016. The results are represented with receiver operator characteristic (ROC) curves and area under ROC curves (AUC) values.
